# How to Treat Asymptomatic and Symptomatic Urinary Tract Infections in the Kidney Transplant Recipients?

**DOI:** 10.7759/cureus.9608

**Published:** 2020-08-07

**Authors:** Zafar Iqbal, Juan Fernando Ortiz, Sawleha Arshi Khan, Amr Salem, Nusrat Jahan

**Affiliations:** 1 Emergency Medicine, California Institute of Behavioral Neurosciences and Psychology, Fairfield, USA; 2 Emergency Department, The Kidney Center, Karachi, PAK; 3 Neurology, California Institute of Behavioral Neurosciences and Psychology, Fairfield, USA; 4 Research, California Institute of Behavioral Neurosciences and Psychology, Fairfield, USA; 5 Hospital Medicine, California Institute of Behavioral Neurosciences and Psychology, Fairfield, USA; 6 Internal Medicine, California Institute of Behavioral Neurosciences and Psychology, Fairfield, USA

**Keywords:** urinary tract infection, kidney transplant recipients, urinary tract infection in kidney transplant recipients

## Abstract

Patients with end-stage renal functions are treated with renal transplantation. After the transplantation, kidney transplant recipients (KTR) are at the risk of urinary tract infection (UTI). UTI in KTR may be symptomatic and asymptomatic. Asymptomatic UTI is the presence of the organisms without any signs and symptoms. There are various ways suggested in the published research papers to deal with UTI in the KTR. The goal of this literature review is to explore how to treat symptomatic and asymptomatic UTI in KTR. A PubMed search was conducted to identify the studies explaining the methods used to deal with UTI in KTR. A total number of 2158 articles were found while searching for regular keywords; however, we found 996 articles with the medical subject heading (Mesh) keywords. After applying the inclusion/ exclusion criteria, 56 articles with the regular keywords search and 29 articles with the Mesh keywords search were selected. These articles included 24 randomized clinical trials, 16 clinical trials, 7 review articles, 5 case reports, 2 controlled clinical trials, 2 observational studies, and 1 cross-sectional study. Our analysis has shown that the early removal of the stent after the transplantation and the use of antibiotics are beneficial in reducing the incidence of symptomatic UTI in the KTR; whereas, treating asymptomatic UTI in KTR has not been proven helpful in reducing the incidence of developing symptomatic UTI later on.

## Introduction and background

The mortality rates in kidney transplant recipients (KTR) are approximately 8.6% within the five years of the transplantation. Among them, 53% are secondary to an infection elsewhere in the body [1]. The incidence of urinary tract infection (UTI) in the renal transplant recipients accounts for 45-72% of all the infections. [2] In the KTR, 30% of all hospitalizations are secondary to UTI [2]. The UTI can be asymptomatic and symptomatic. The asymptomatic UTI is defined as the presence of the organism; however, there are no signs and symptoms and it accounts for 17-51% of infections in the KTR and risking the individuals for the subsequent UTI [3].

Several articles have been published in the past that have shown the incidence of UTI in the KTR and various ways to prevent it from occurring. Whether to treat KTR suffering from asymptomatic UTI with the antimicrobials and the impacts of antimicrobials therapy or dealing with the urinary catheters in individuals with symptomatic UTI after the transplantation have been discussed in these articles [4,5]. The knowledge of dealing with the asymptomatic and the symptomatic UTI in the KTR is important in order to improve the health of these individuals, minimize the incidence of hospital admission, and reduce the financial burden on the health sector.

The aim of this literature review is to evaluate the available data in order to find the measures for the treatment of asymptomatic and symptomatic UTI in KTR.

## Review

Method

The available literature was searched in PubMed with regular keywords and medical subject heading (MeSH) subheadings to collect data. 

Regular keywords for literature search included urinary tract infection in kidney transplant recipients, urinary tract infections, and kidney transplant recipients. While searching for the regular keywords UTI in KTR, UTI, and KTR, a total number of 2158 articles were found. Out of them, 56 articles were selected for this review. 

The literature was also searched for MeSH keywords. MeSH keywords for literature search included UTI in KTR, UTI, and KTR. This search found 996 records and 29 of them were included in this study. 

Studies were selected after applying the inclusion/exclusion criteria. Inclusion criteria involved studies on clinical trials, controlled clinical trials, randomized clinical trials, observational study, reviews, systemic reviews, human studies, papers published in the English language, and case reports. However, exclusion criteria involved studies meta-analysis, animal studies, and papers published in other than the English language.

Results

Table 1 shows the total number of articles found while searching for the regular keywords UTI in KTR, UTIs, and KTR. A record of 2158 articles was found, and after applying the inclusion/exclusion criteria, 56 articles were selected and reviewed. 

**Table 1:** Total number of articles after applying inclusion/exclusion criteria

Regular keywords: Urinary tract infection in kidney transplant recipients, urinary tract infection, kidney transplant recipients	
Total records	2158
English	1895
Human	1682
Systematic reviews	315
Review	313
Observational study	109
Clinical trials	89
Controlled clinical trial	56
Randomized controlled trial	56

Table 2 shows the total number of articles found while searching for the MeSH keywords UTI in KTR, UTIs, and KTR. This search found 996 articles. After applying the inclusion/exclusion criteria, 29 articles were selected and reviewed. 

**Table 2:** Total number of articles after applying inclusion/exclusion criteria

MeSH keywords: Urinary tract infection in kidney transplant recipients, urinary tract infection, kidney transplant recipients	
Total records	996
English	852
Human	847
Systematic reviews	146
Review	145
Observational study	55
Clinical trials	43
Controlled clinical trial	29
Randomized controlled trial	29

Overall, a total number of 85 articles were selected from the regular and MeSH keywords search UTI, UTI in KTR, KTR, and reviewed. Rests of the 3069 articles were removed due to one of the reasons not specifying the disease of interest, meta-analysis, animal studies, and the paper published in other than the English language. All the available records reviewed were free including the citations. A manual collection of data was done after reviewing individual articles and applying inclusion/exclusion criteria in order to include the relevant articles.

Discussion

In the analysis of the 57 published articles, we found out that the most common bacterial infection in KTR is UTI and the majority of the causative organisms found included Gram-negative (76%) with Escherichia coli (33%) and Enterococcus and Klebsiella enterobacter (20%) [6]. The predisposing factors for the UTI in the KTR are indwelling catheters, anatomical defects, neurogenic bladder, rejection, traumatic injury to the renal system, and immunosuppressant [6]. Identification and treatment of the risk factors are crucial in order to reduce the incidence of UTI in the KTR. This analysis focused on the various ways adopted to deal with the number of asymptomatic and symptomatic UTI in KTR.

The presence of ureteric stents and catheters in the KTR predisposes them to UTI. Table 3 shows that the removal of these ureteric stents and catheters earlier can play a pivotal role in reducing the incidence of UTI in the KTR. On average, the removal of ureteric stents and catheters as early as one to two weeks after transplantation has been associated with fewer incidences of UTIs compared to the removal of UTIs beyond two weeks.

**Table 3:** Summary of studies showing the effect of early removal of the ureteric stent/catheters earlier in reducing the incidence of UTI in the KTR UTI: urinary tract infection, KTR: kidney transplant recipient.

Author/date	Study design	Population	Sample size	Main points	P-value
Patelet al. [7]	Randomized controlled trial	Patients aged 2-75 years undergoing renal transplantation in the six UK hospitals.	205	After renal transplantation, late removal of the stent at week 6 had a higher incidence of UTI 31 out of 126 subjects (24.6%) compared to early removal at day 5, 6 of 79 (7.6%).	P = 0.004
Huang et al. [8]	Randomized controlled trial	Two groups based on the duration of double-J stent placement was, group 1 six weeks and group 2 three weeks. Group 1 included 186 patients and group 2 included 179 patients.	365	Compared to group 1, patients in group 2 had fewer UTI episodes.	P = NA
Parapiboon et al. [9]	Randomized controlled trial	Ureteric stent removal eight days (37 patients) or routine ureteric stent removal 15 days (37 patients) after kidney transplantation in Thailand from April 2010 to January 2011.	74	The incidence of UTI in early stent removal was less than the routine stent removal group (15/37, 40.5% vs 27/37, 72.9%)	P = 0.004
Tavakoli et al. [10]	Randomized controlled trial	Patients receiving a renal transplant were randomized preoperatively to undergo double-J stent (112) or no-stent (89) ureterovesical anastomosis from November 1998 to October 2001.	201	Higher incidence of UTI in patients with stent in situ for >30 days after transplantation compared to the rate in those with no stent.	P < 0.02
Liuet al. [11]	Prospective, randomized clinical trial	Between October 2010 and March 2015 patients with early ureteral stent removal at week 1 (52) for group 1 or the routine ureteral stent removal at week 4 (51) for group 2 in KTR.	103	Three episodes of UTIs occurred in the 1-week stent group, and 18 such episodes were recorded in the 4-week stent group 5.8% vs 29.4%	P = 0.002
Luján et al. [12]	Controlled clinical trial	111 patients with double J catheter and another of 83 catheter-free patients.	194	No differences between the two groups in regards to UTI 3 (2.7%) in the catheter group and 1 (1.2%) in the catheter-free group.	P = 0.63
Menezes et al. [5]	Randomized controlled trial	From March 2013 to December 2014, a randomized 1:1 ratio through computer-generated system to a nitrofurazone-coated silicone urinary catheter and non-impregnated silicone urinary catheter in São Paulo, Brazil.	176	No differences noted in the rates of UTI (8% in the nitrofurazone group and 6.8% in the control group.	P = 0.99

The role of antimicrobials in the control of the incidence rate of UTI in the KTR has also been discussed in these papers. The published studies have shown that the perioperative and postoperative use of antibiotics has beneficial effects on individuals undergoing renal transplantation. Table 4 demonstrates the impacts of antimicrobial use on the occurrence of UTI in KTR. In summary, individuals undergoing kidney transplants treated with antibiotics in the perioperative and postoperative periods had a lower incidence of UTI relative to those individuals who did not receive any antimicrobials; however, the choice of antimicrobials differs.

**Table 4:** Impact of antimicrobials use over the incidence of UTI in KTR KTR: kidney transplant recipient, UTI: urinary tract infection, TMP/SXT: trimethoprim-sulfamethoxazole, FOS: fosfomycin trometamol.

Author/date	Study design	Population	Sample size	Main points	P-value
Lee et al. [13]	Clinical trial	Adult KTR between September 2014 and December 2015.	277	56 recipients (20%) received additional antibiotic prophylaxis (ABX+) and 221 (80%) did not (ABX-) at the time of ureteral stent removal. The difference in the occurrence of UTI in the ABX(+) group (16%) and ABX(-) group (19%) was insignificant.	P = 0.85
Arreola-Guerra et al. [4]	Randomized controlled trial	The intervention group including 32 patients who received 3 g of FOS PO every 10 days and TMP-SMX (160/800 mg) three times per week (group 1), whereas the control group including 35 patients who received TMP-SMX (160/800 mg) daily (group 2).	67	The incidence of UTI in group 1 vs group 2 was 40.6% vs 42.8%.	P = 0.85
Salehipour et al. [14]	Randomized controlled trial	The bladders of the group 1, the amikacin group, were filled with a saline solution containing amikacin (1 g in adults and 30 mg/kg in pediatric patients), whereas the bladders of the patients of the group 2, the control group, were filled with a saline solution and were followed up for three months after transplantation.	200	The overall incidence of UTIs was found to be significantly lower in the amikacin group (25 vs. 49%).	P = 0.0007
Hibberd et al. [15]	A double-blind, randomized controlled trial	To compare low-dose TMP/SXT with ciprofloxacin for the prevention of UTI in KTR. Patients received either ciprofloxacin (250 mg), 51 patients or TMP/SXT (80 mg trimethoprim, 400 mg sulfamethoxazole) 52 patients daily for six months following transplantation.	103	Treatment was successful in 75% (38 of 51) receiving ciprofloxacin and 71% (37 of 52) treated with TMP/SXT. Ciprofloxacin is at least as effective as TMP/SXT.	P = 0.87
Khosroshahi et al. [16]	Randomized controlled trial	The efficacy of various doses of TMP/SXT was observed in group 1 (n = 63) who received low to moderate doses of TMP/SXT (either 80/400 mg or 160/800 mg, daily) and group 2 (n = 32) who received high doses of TMP/SXT (320/1600 mg), daily in two divided doses.	95	UTI was observed in about 25% of patients on the high-dose versus 49.2% of those on low- to moderate-dose prophylaxis.	P < 0.05

The data have also been analyzed in various ways to deal with the asymptomatic UTI in the KTR. The studied papers found that the treatment of asymptomatic urinary tract infection in post-transplant individuals has not been shown to be beneficial in preventing symptomatic UTI at a later point. Table 5 summarizes the incidence studies on how to deal with asymptomatic UTI in individuals with kidney transplants.

**Table 5:** Summarizes the studies showing the incidence and how to deal with asymptomatic UTI KTR: kidney transplant recipient, UTI: urinary tract infection, AB: asymptomatic bacteriuria.

Author/date	Study design	Population	Sample size	Main points	P-value
Origuen et al. [17]	Randomized controlled trial	Between January 2011 and December 2013, patients developing one or more episodes of AB beyond the second after transplantation were included in this open-label trial. Participants were randomized (1:1 ratio) to the treatment group (systematic antimicrobial therapy for all episodes of AB occurring ≤24 months after transplantation [53 patients]) or control group (no antimicrobial therapy [59 patients]).	112	No significant differences noted in the incidence of UTI	P = 1.00
Julien Coussementet al. [18]	Cross-sectional study	KTR undergoing routine surveillance in three outpatient transplant clinics in Belgium and France. Patients who were in the first two months post-transplantation and/or had a urinary catheter excluded. Asymptomatic participants who had a urine culture with one organism isolated at ≥105 CFU/mL were asked to provide a confirmatory urine specimen.	500	Overall, the prevalence of AB was 3.4% (17/500 patients). It was similarly low among KTR who were between 2 and 12 months after transplantation (1.3%, 1/76 patients) and those who were farther after transplantation (3.8%, 16/424 patients: P = 0.49).	P = 0.49

The outcome of the study was removing stent earlier after the transplantation which reduces the incidences of getting UTI in KTR patients. Studies have shown that removing these devices one to two weeks after the transplantation has fewer chances of UTI in these individuals as compared to removing it beyond the two weeks period. Similarly, the use of prophylactic antibiotics in the perioperative and postoperative phases has beneficial effects in reducing the occurrence of UTI in these patients. However, the treatment of asymptomatic UTI may not have any beneficial effects to avoid the occurrence of symptomatic UTI at a later stage. More work is needed to find out why the treatment of asymptomatic UTIs has not been associated with any positive outcome in the KTR. There are some limitations to the current literature review: not including papers published other than English, meta-analysis results, animal studies, and many other unexplored variables that can be tested in future studies. The outcome could be different if the limitations have been included.

## Conclusions

In the analysis of 57 published articles, we found out that the early removal of the devices including the ureteric stents and catheters in the post-kidney transplant period and the perioperative and postoperative antimicrobials therapies have been proven beneficial in reducing the chances of getting UTI in KTR. However, this study also found out that treating asymptomatic UTI in KTR has no positive impact in reducing the incidence of symptomatic UTI in KTR later on. More research is needed with a larger cohort and prospective randomized control trials with drugs and placebo to get the answer to this research question that why treating asymptomatic UTI in KTR with the antimicrobials has no benefits.
